# Systemic Drug Prophylaxis and Treatment of Sympathetic Ophthalmitis

**Published:** 1911-03

**Authors:** 


### Systemic Drug Prophylaxis and Treatment of Sympathetic Ophthalmitis

SINCE the announcement by Dr. Wilfred M. Barton of Washington, D. C., (*Journal of the A.M.A.,* March 12, 1909), that urotropin given per os is promptly excreted by the mucus membranes of the middle ear, the suggestions for the employment of this drug in other suppurative and inflammatory affections based on its appearance in the excretions and its ability to render them sterile, have followed each other rapidly.

The most recent of these is contained in a preliminary communication to the *Journal of Ophthalmology and Oto-Laryngology*, Nov., 1910, by Dr. M. R. Dinkelspiel of Wilkesbarre, Pa., late instructor in ophthalmology and assistant to hospital dispensary staff, Medico-Chirurgical College, Philadelphia. The author has obtained striking results in a series of consecutive cases of sympathetic ophthalmitis and other affections of the uveal tract by the administration per os of 40 to 80 grains of urotropin daily, in 10 grain doses. Under this treatment he has observed sympathetic ophthalmitis and iridocyclitis associated with hypopyon clear up without any other constitutional medication except an occasional purgative.

Whether sympathetic ophthalmitis is caused through the blood stream or by continuity through the brain the drug can probably reach the course of invasion by either route. Further experiments are now in process of conduction and more clinical data will, of course, be necessary to give the treatment a definite therapeutic position.

